# SIPIBEL observatory: Data on usual pollutants (solids, organic matter, nutrients, ions) and micropollutants (pharmaceuticals, surfactants, metals), biological and ecotoxicity indicators in hospital and urban wastewater, in treated effluent and sludge from wastewater treatment plant, and in surface and groundwater

**DOI:** 10.1016/j.dib.2021.107726

**Published:** 2021-12-16

**Authors:** Jean-Luc Bertrand-Krajewski, Rémy Bournique, Vivien Lecomte, Noémie Pernin, Laure Wiest, Christine Bazin, Agnès Bouchez, Elodie Brelot, Benoît Cournoyer, Teofana Chonova, Christophe Dagot, Pascal Di Majo, Adriana Gonzalez-Ospina, Audrey Klein, Jérôme Labanowski, Yves Lévi, Yves Perrodin, Sandra Rabello-Vargas, Liana Reuilly, Audrey Roch, Axel Wahl

**Affiliations:** a Univ Lyon, INSA Lyon, DEEP, EA 7429, 11 rue de la physique, Villeurbanne Cedex F-69621, France; b Groupe de Recherche, d'Animation technique et d'Information sur l'Eau (GRAIE), 66 boulevard Niels Bohr, Villeurbanne F-69100, France; c Univ Lyon, CNRS, Université Claude Bernard Lyon 1, ENS de Lyon, Institut des Sciences Analytiques, UMR 5280, 5 rue de la Doua, Villeurbanne F-69100, France; d Provademse, CEI 1 Building, 66 boulevard Niels Bohr, Villeurbanne F-69100, France; e INRAE, USMB, UMR CARRTEL, 85 bis avenue de Corzent, Thonon-les-Bains F-74200, France; f UMR Ecologie microbienne, CNRS 5557, INRA 1418, UCBL, VetAgro Sup, Bâtiment principal, aile 3, 1er étage, Marcy l’Étoile F-69280, France; g Université de Limoges, RESINFIT, UMR-Inserm 1092, 2 rue du Dr Marcland, Limoges F-87000, France; h Centre Hospitalier Alpes Léman (CHAL), 558 route de Findrol, BP 20 500, Contamine-sur-Arve F-74130, France; i Wastewater Technical & Innovation Division, Suez – Treatment Infrastructure, 183 avenue du 18 Juin 1940, Rueil-Malmaison F-92500, France; j Commission Internationale pour la Protection des Eaux du Léman (CIPEL), 50 route de Duillier, CP 1080, Nyon CH-1260, Switzerland; k Université de Poitiers, ENSIP, UMR CNRS 7285, IC2MP, 1 rue Marcel Doré, Poitiers Cedex F-86073, France; l Université Paris Sud, Université Paris-Saclay, UMR 8079, CNRS, AgroParisTech, Faculté de Pharmacie, 5 rue Jean-Baptiste Clément, Chatenay-Malabry F-92290, France; m Univ Lyon, ENTPE, CNRS, UMR 5023 LEHNA, rue Maurice Audin, Vaulx-en-Velin Cedex F-69518, France; n Service de l'écologie de l'eau, Etat de Genève, 25 avenue de Sainte-Clotilde, Genève CH-1211, Switzerland; o Syndicat Mixte d'Aménagement de l'Arve et de ses Abords, 300 chemin des Prés Moulin, Saint-Pierre-en-Faucigny F-74800, France; p Syndicat des eaux des Rocailles et de Bellecombe, 160 Grande Rue, Reignier-Esery F-74930, France; q Services Industriels de Genève, Chemin du Château-Bloch 2, Vernier CH-1219, Switzerland

**Keywords:** Hospital wastewater, urban wastewater, emerging contaminants, ecotoxicological risk assessment, wastewater treatment plant, sludge, receiving water body, groundwater

## Abstract

The Bellecombe pilot site – SIPIBEL – was created in 2010 in order to study the characterisation, treatability and impacts of hospital effluents in an urban wastewater treatment plant. This pilot site is composed of: i) the Alpes Léman hospital (CHAL), opened in February 2012, ii) the Bellecombe wastewater treatment plant, with two separate treatment lines allowing to fully separate the hospital wastewater and the urban wastewater, and iii) the Arve River as the receiving water body and a tributary of the Rhône River and the Geneva aquifer. The database includes in total 48 439 values measured on 961 samples (raw and treated hospital and urban wastewater, activated sludge in aeration tanks, dried sludge after dewatering, river and groundwater, and a few additional campaigns in aerobic and anaerobic sewers) with 44 455 physico-chemistry values (including 15 pharmaceuticals and 14 related transformation products, biocides compounds, metals, organic micropollutants), 2 193 bioassay values (ecotoxicity), 1 679 microbiology values (including microorganisms and antibioresistance indicators) and 112 hydrobiology values.

## Specifications Table

**Table:** 

Subject	Environmental Engineering
Specific subject area	Pollutants, pharmaceuticals, surfactants, biocides, biological and ecotoxicity indicators in wastewater, river, groundwater and sludge
Type of data	Tables
How data were acquired	Data have been obtained applying ISO, EN, French and XP (experimental) standard methods, or laboratories’ internal methods. These methods are listed and described in four csv files of the Sipibel database available on Zenodo (https://doi.org/10.5281/zenodo.5176067): 04_SIPIBEL_metadata_physico_chemistry_methods.csv 05_SIPIBEL_metadata_bioassay_methods.csv 06_SIPIBEL_metadata_microbiology_methods.csv 07_SIPIBEL_metadata_hydrobiology_methods.csv
Data format	Validated raw data in CSV files
Parameters for data collection	More than 200 monitored quantities: • Physico-chemistry (dissolved and particulate fractions) - Usual indicators (pH, TSS, COD, BOD_5_, TOC, N, P, S, ions…) - Micropollutants: 15 pharmaceuticals and 14 transformation products, 9 metals or metalloids, surfactants (anionic, cationic, alkylphenols, LAS…), AOX, PAHs, organohalogenic compounds • Microbiology - Resistance integrons (Class 1, 2, and 3 integrons) - *Pseudomonas aeruginosa* (opportunistic pathogens) • Endocrine disruption potential: oestrogenic and thyroid disrupting effects • Ecotoxicology - Bioassays with micro-crustaceans (*Daphnia magna, Heterocypris incongruens*), rotifer (*Brachionus calyciflorus)* and micro-algae (*Pseudokirchneriella subcapitata*) - Genotoxicity tests: Comets assay and SOS Chromotest • Hydrobiology - NGBI (Normalised Global Biological Index) - DBI (Diatom Biological Index) Each value in the dataset is associated with the analytical method used, as well as with the conditions at the time of the sampling (date and duration of the sampling). Flow rates are also provided to calculate loads.
Description of data collection	The data were acquired at different locations: • Hospital and urban inlets of the Bellecombe wastewater treatment plant (WWTP) • Hospital and urban outlets of the Bellecombe WWTP • Outlet of the Arcachon hospital • Outlets of the Ocybele and Villette WWTPs • Inlet and outlet of the Choully combined sewer gallery • Arve and Rhône Rivers, upstream and downstream the WWTPs • Vessy groundwater recharge station • Geneva aquifer The data were collected during several sampling campaigns on each site, from 2011 to 2017, at different periods. Water samples were collected with auto-samplers and sludge samples were collected manually. See Section 4 for details.
Data source location	• Arve River catchment, Haute-Savoie, France • Geneva region, Switzerland and France • Arcachon, Gironde, France GPS coordinates of sampling points: see Fig. 1 and Table 1.
Data accessibility	Eleven files are available on Zenodo in the SIPIBEL dataset zip file: - the SIPIBEL data set description (one PDF file); - the SIPIBEL metadata (five CSV files: 1) sampling points, 2) physico-chemistry methods, 3) bioassay methods, 4) microbiology methods, and 5) hydrobiology methods); - the SIPIBEL data sets (five CSV files: 1) samples, 2) physico-chemistry data, 3) bioassay data, 4) microbiology data, and 5) hydrobiology data). Data set doi: 10.5281/zenodo.5176067.
Related publications	Selected publications presenting and/or using the SIPIBEL data: ­ Bertrand-Krajewski, J.-L. Pharmaceuticals and detergents in hospital and urban wastewater: comparative monitoring, treatment, and assessment of impacts. *Environ Sci Pollut Res* **25,** 9195–9196 (2018). https://doi.org/10.1007/s11356-018-1445-0. ­ Chonova, T., Lecomte, V., Bertrand-Krajewski, J.-L. *et al*. The SIPIBEL project: treatment of hospital and urban wastewater in a conventional urban wastewater treatment plant. *Environ Sci Pollut Res* **25**, 9197–9206 (2018). https://doi.org/10.1007/s11356-017-9302-0.
	­ Wiest, L., Chonova, T., Bergé, A. *et al.* Two-year survey of specific hospital wastewater treatment and its impact on pharmaceutical discharges. *Environ Sci Pollut Res* **25,** 9207–9218 (2018). https://doi.org/10.1007/s11356-017-9662-5. ­ Bergé, A., Wiest, L., Baudot, R. *et al*. Occurrence of multi-class surfactants in urban wastewater: contribution of a healthcare facility to the pollution transported into the sewerage system. *Environ Sci Pollut Res* **25**, 9219–9229 (2018). https://doi.org/10.1007/s11356-017-0470-8. ­ Kiss, A., Bergé, A., Domenjoud, B. *et al.* Chemometric and high-resolution mass spectrometry tools for the characterization and comparison of raw and treated wastewater samples of a pilot plant on the SIPIBEL site. *Environ Sci Pollut Res* **25,** 9230–9242 (2018). https://doi.org/10.1007/s11356-017-0748-x. ­ Laquaz, M., Dagot, C., Bazin, C. *et al.* Ecotoxicity and antibiotic resistance of a mixture of hospital and urban sewage in a wastewater treatment plant. *Environ Sci Pollut Res* **25,** 9243–9253 (2018). https://doi.org/10.1007/s11356-017-9957-6. ­ Chonova, T., Labanowski, J., Cournoyer, B. *et al.* River biofilm community changes related to pharmaceutical loads emitted by a wastewater treatment plant. *Environ Sci Pollut Res* **25,** 9254–9264 (2018). https://doi.org/10.1007/s11356-017-0024-0. ­ Pouzol T., Lévi Y., Bertrand-Krajewski J.-L. (2020). Modelling daily and hourly loads of pharmaceuticals in urban wastewater. *Int. J. Hyg. Environ. Health*, **229**, 20 p. doi.org/10.1016/j.ijheh.2020.113552. Available online 11 June 2020. ­ Buelow E., Rico A., Gaschet M, LourencoJ., Kennedy S.P., Wiest L., Ploy MC., Dagot C. (2020). Hospital discharges in urban sanitation systems: long-term monitoring of wastewater resistome and microbiota in relationship to their eco-exposome. *Wat. Res. X*, 7, 100045. https://doi.org/10.1016/j.wroa.2020.100045. In addition, all publications and SIPIBEL project deliverables, in French or English, are listed in the SIPIBEL website at http://www.graie.org/Sipibel/publications.html (accessed 05 July 2021). Note that the SIPIBEL website is in French, except the welcome page at http://www.graie.org/Sipibel/anglais.html.

## Value of the Data

• The very large SIPIBEL data set contains values of numerous indicators (usual physico-chemical indicators of water pollution, metals and chemical elements, pharmaceuticals, PAHs, biocides, bioassays, integrons and antibiotic resistance, etc.) measured, in a coordinated way, in (i) hospital and urban wastewater collection systems, (ii) a wastewater treatment plant (both water and sludge treatment lines), and (iii) receiving water bodies at local and regional scales, allowing a global anaysis of concentrations and loads from emissions to discharges into the environment. The interest of this data set also lies in its spatio-temporal dynamics with an evolution of the successive configurations of the wastewater treatment plant (Fig. 2) evaluated over 4 years of monitoring.
• In particular, these data contribute to a better assessment of (i) concentrations and loads of pharmaceuticals and biocides in urban and hospital wastewater, (ii) their removal by wastewater treatment plants, (iii) the validation of indicators like integrons or antibioresistance in environment, and (iv) the influence of exposome on resistome.
• These data can be used by researchers working on pharmaceuticals and biocides in wastewater, in treament plants and in receiving surface water bodies. They can also be used by wastewater treatment plant staff, consultants, or water utilities to design future treatment processes, and also by regulators to inform their decisions about new policies and regulations related to pharmaceuticals and biocides in wastewater and in the environment. For example, these data have been used to establish correlation by machine learning system or to model the antibioresistance dissemination.
• The data can also be used in modelling works, and in international reviews and comparisons.

## 1 Data

This data paper presents the results of a 7-year study (2011-2017) at the Bellecombe pilot site - hereafter SIPIBEL - that aims at the characterisation, treatability and impacts of hospital effluents in an urban wastewater treatment plant [1].

The SIPIBEL observatory provides data on both (i) conventional water quality parameters, and (ii) pharmaceuticals, transformation products and surfactants presence, and (iii) biological indicators that enable the long-term evaluation of risks for the environment and health:

• Physico – Chemistry (PC):
  ○ Standard indicators: pH, conductivity, BOD_5_, COD (+ residual COD + soluble COD), organic fraction, mineral fraction, DOC, organic carbon, TOC, sulphates, sulphides, TSS (+ organic TSS + mineral TSS), NO_2_^−^, NO_3_^−^, NH_4_^+^, TKN, residual TKN, total phosphorus, PO_4_^3−^, C/N ratio, dry content
  ○ Metals and metalloids:
    ■ Arsenic (As)
    ■ Cadmium (Cd)
    ■ Chrome (Cr)
    ■ Copper (Cu)
    ■ Gadolinium (Gd)
    ■ Lead (Pb)
    ■ Mercury (Hg)
    ■ Nickel (Ni)
    ■ Zinc (Zn)
  ○ Pharmaceuticals and metabolites:
    ■ Analgesic: paracetamol, salicylic acid
    ■ Anti-inflammatory: ibuprofen, ketoprofen, diclofenac
    ■ Antihypertensive: atenolol, propranolol
    ■ Hormonal contraceptive: ethinylestradiol
    ■ Antibiotic: ciprofloxacin, sulfamethoxazole, vancomycin
    ■ Anti-fungal: econazole
    ■ Anti-convulsant: carbamazepine
    ■ Four metabolites of sulfamethoxazole: N4-acetyl-sulfamethoxazole (SMX impurity A), Sulfamethoxazole B-D-Glucuronide (SMX glucuronide), 4-Amino-N-(3-methyl-5-isoxazolyl)benzenesulfonamide (SMX impurity F), 4-Nitro-sulfamethoxazole (SMX-NO2)
    ■ Nine metabolites of diclofenac: 4-hydroxy diclofenac (DCF 4HO), 5-hydroxy diclofenac (DCF 5HO), Diclofenac carboxylic acid (DCF COOH), Diclofenac acyl-B-D-glucuronide (DCF glucuronide), 2-[(2-chlorophenyl)-amino] benzaldehyde (DCF CPAB), 2-[(2,6-Dichlorophenyl)amino]benzaldehyde (DCF impurity B), 1-(2,6-Dichlorophenyl)-1,3-dihydro-2H-indol-2-one (DCF lactam), [2-[(2,6-Dichlorophenyl)amino]phenyl]methanol (DCF impurity C), 1,3-Dihydro-2H-indol-2-one (DCF impurity E)
  ○ Surfactants:
    ■ Total anionic surfactants
    ■ Total cationic surfactants
    ■ Total non-ionic surfactants
    ■ Alkylphenols: 4-tert-octylphenol, 4-n-nonylphenol, nonylphenol monoethoxylate, nonylphenol diethoxylate
    ■ Other surfactants: anionic (Sodium 2-ethylhexyl sulfate; Sodium dodecyl sulfate (SDS); Sodium laureth sulfate (Texapon N 701 S); LAS (linear alkylbenzene sulfonate) C10-C13), cationic (benzyldimethyldodecylammonium chloride (BDDAC or BAC C12); benzyldimethyltetradecylammonium chloride (BDTAC or BAC C14); Stepanquat GA 90; Incromine SD; Lauryl pyridinium chloride), zwitterionic (Cetyl Betaïne), non-ionic (Comperlan 100 (Cocamide MEA); Triton X-100) and Benzotriazole
  ○ Organohalogenated compounds: 1,2-dichloroethane, 1,1-dichloroethane, 1,1-dichloroethylene, 1,2-dichloroethylene (trans), 1,2-dichloroethylene (cis), Hexachloroethane, 1,1,2,2-tetrachloroethane, 1,1,2-trichlorotrifluoroethane, 1,1,1-trichloroethane, 1,1,2-trichloroethane, Vinyl chloride, 1,2-dibromoethane, 1,2-dichloropropane, Bromochloromethane, Chloroform, Bromoform, Methyl chloride, 3-chloropropene, 2,3-dichloropropene, Cis-1,3-dichloropropylene, Trans-1,3-dichloropropylene, Dibromochloromethane, Dichloromethane, Dichlorobromomethane, Tetrachloroethylene, Carbon tetrachloride, Trichloroethylene, Hexachlorobutadiene, total AOX
  ○ Polycyclic aromatic hydrocarbons (PAHs): Anthracene, Fluoranthene, Naphthalene, Benzo(a)pyrene, Benzo(b)fluoranthene, Benzo(k)fluoranthene, Benzo(ghi)perylene, Indeno(1,2,3,c,d)pyrene
• Microbiology
  ○ Resistance integrons (Class 1, 2, and 3 integrons)
  ○ *Pseudomonas aeruginosa* (opportunistic pathogens)
• Endocrine disruption potential: oestrogenic and thyroid disrupting effects
• Ecotoxicology
  ○ Bioassays with micro-crustaceans (*Daphnia magna, Heterocypris incongruens*), rotifer (*Brachionus calyciflorus)* and micro-algae (*Pseudokirchneriella subcapitata*)
  ○ Genotoxicity tests: Comets assay and SOS Chromotest
• Hydrobiology
  ○ NGBI (Normalised Global Biological Index)
  ○ BID (Diatom Biological Index).

Each value in the dataset is associated with metadata: sampling point, analytical or measurement method, LoD (limit of detection), LoQ (limit of quantification), validation mark, date and time of sampling campaigns, etc. Discharges at some sampling points are also provided to calculate loads.

In case of substances detected at concentrations lower than their limit of quantification (< LoQ) or lower than their limit of detection (< LoD), the following substitutions were applied, respectively, according to the frequency of quantification (FQ) of the substance [3]:

If FQ < 25%, < LoQ was substituted with 0 and < LoD was substituted with 0.
If 25% < FQ < 50%, < LoQ was substituted with LoQ/4 and < LoD was substituted with LoD/4.
If 50% < FQ < 75%, < LoQ was substituted with LoQ/2 and < LoD was substituted with LoD/2.
If FQ > 75%, < LoQ was substituted with LoQ and < LoD was substituted with LoD.

In addition, for very rare detections, if less than 5 values have been detected and measured, < LoQ was substituted with 0 and < LoD was substituted with 0.

The LoQ values for all physico-chemical and microbiological methods are given in two csv files of the Sipibel database available on Zenodo (https://doi.org/10.5281/zenodo.5176067): 04_SIPIBEL_metadata_physico_chemistry_methods.csv and 06_SIPIBEL_metadata_microbiology_methods.csv.

## 2 Experimental Design, Materials and Methods

### 2.1 General description

**Fig. 1:**
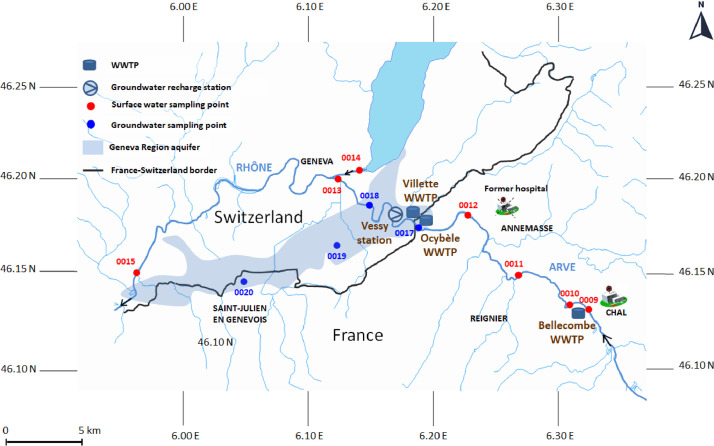
Schematic map of SIPIBEL sampling points (SPs) locations. See Table 1 for detailed GPS coordinates.

SIPIBEL is located in the Arve River basin, in France, close to the French – Swiss border (Fig. 1). The pilot site includes the following elements:

• The CHAL hospital, opened in February 2012, with 450 beds, 8 surgery rooms, and various departments, including emergency, oncology, nuclear medicine diagnosis, internal medical labs, pharmacy, and kitchen.
• The Bellecombe activated sludge WWTP, with the possibility to treat either separately or jointly the CHAL wastewater and the urban wastewater from the Bellecombe urban catchment (approx. 21 000 inhabitants).
• The Arve River, downstream the Bellecombe WWTP, flowing from the French Alps into the Rhône River. The Geneva aquifer is used for drinking water production by both France and Switzerland. As the groundwater intake is greater than the natural recharge, the Vessy station injects Arve River water after river bank filtration to recharge the aquifer (the mean annual injected volume is approximately 9 million m^3^, which is significant as it amounts to 60% of the annual groundwater intake for drinking water production).

The initial capacity of the Bellecombe activated sludge WWTP was 5 400 PE (aeration tank 1 280 m^3^, treatment line F2). It was enlarged for the first time in 1995 with a second basin (10 600 PE — 2 720 m^3^, tratment line F1). In 2009, a third basin was constructed (16 000 PE — 4 000 m^3^, treatment line F3) which led to a total capacity of 32 000 PE. The last extension was performed due to the connection of the CHAL hospital. The urban combined sewer network connected to the WWTP collects the urban wastewater (UWW) of approximately 21 000 inhabitants. The hospital wastewater (HWW) was initially estimated at 2 000 PE. It is transferred without specific pretreatment (except iodine decay tanks located in the CHAL basement for the treatment of radioactive urine from a few rooms with patients treated for cancer) by a separate sewer system to the WWTP.

The Bellecombe WWTP is equipped with pre-treatment bar screens and aerated grit chambers. The wastewater then enters into basins with activated sludge operating sequentially under aerobic and anoxic conditions. Subsequently, the treated wastewater is pumped into a final clarifier for sludge separation before discharge into the Arve River.

**Fig. 2:**
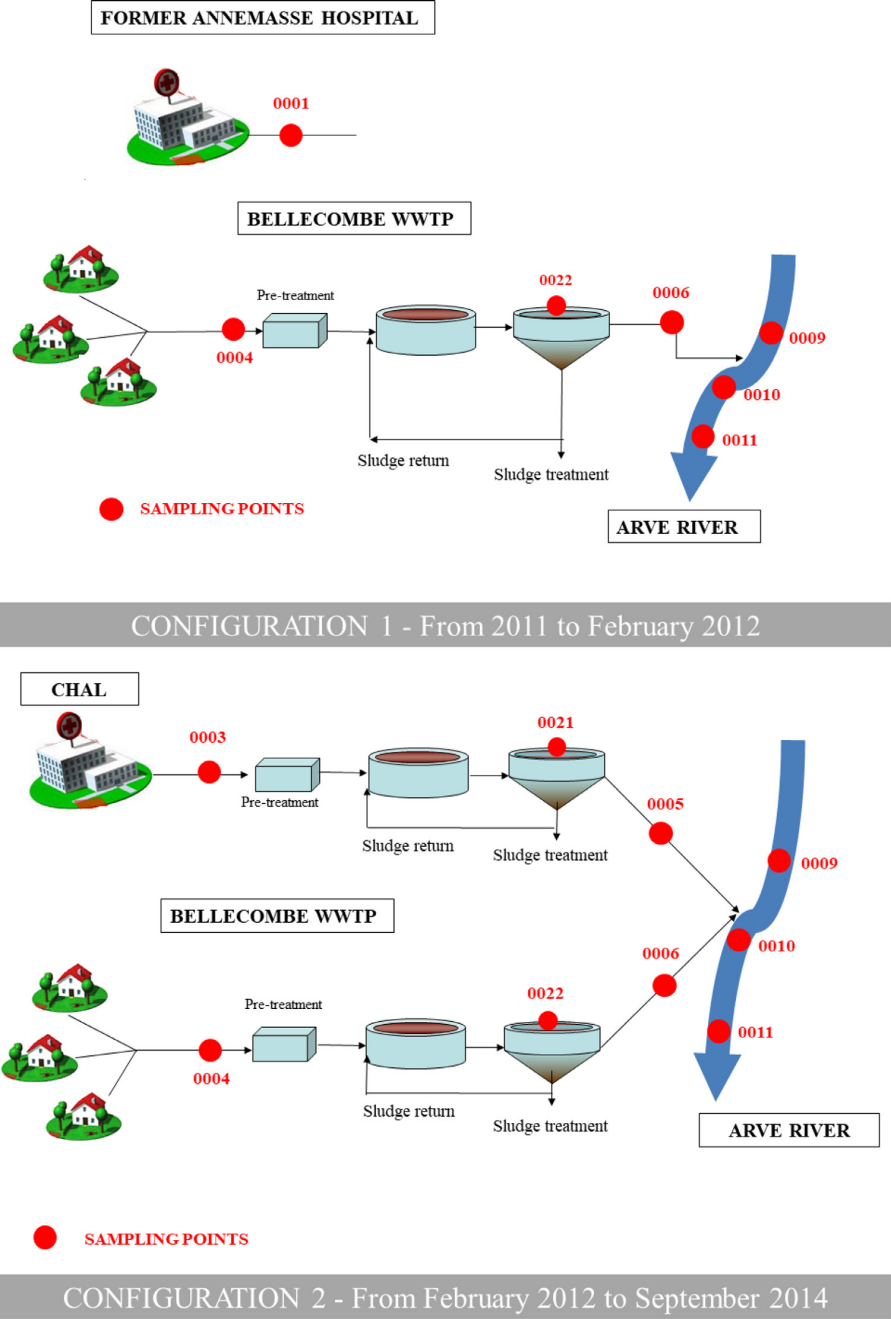

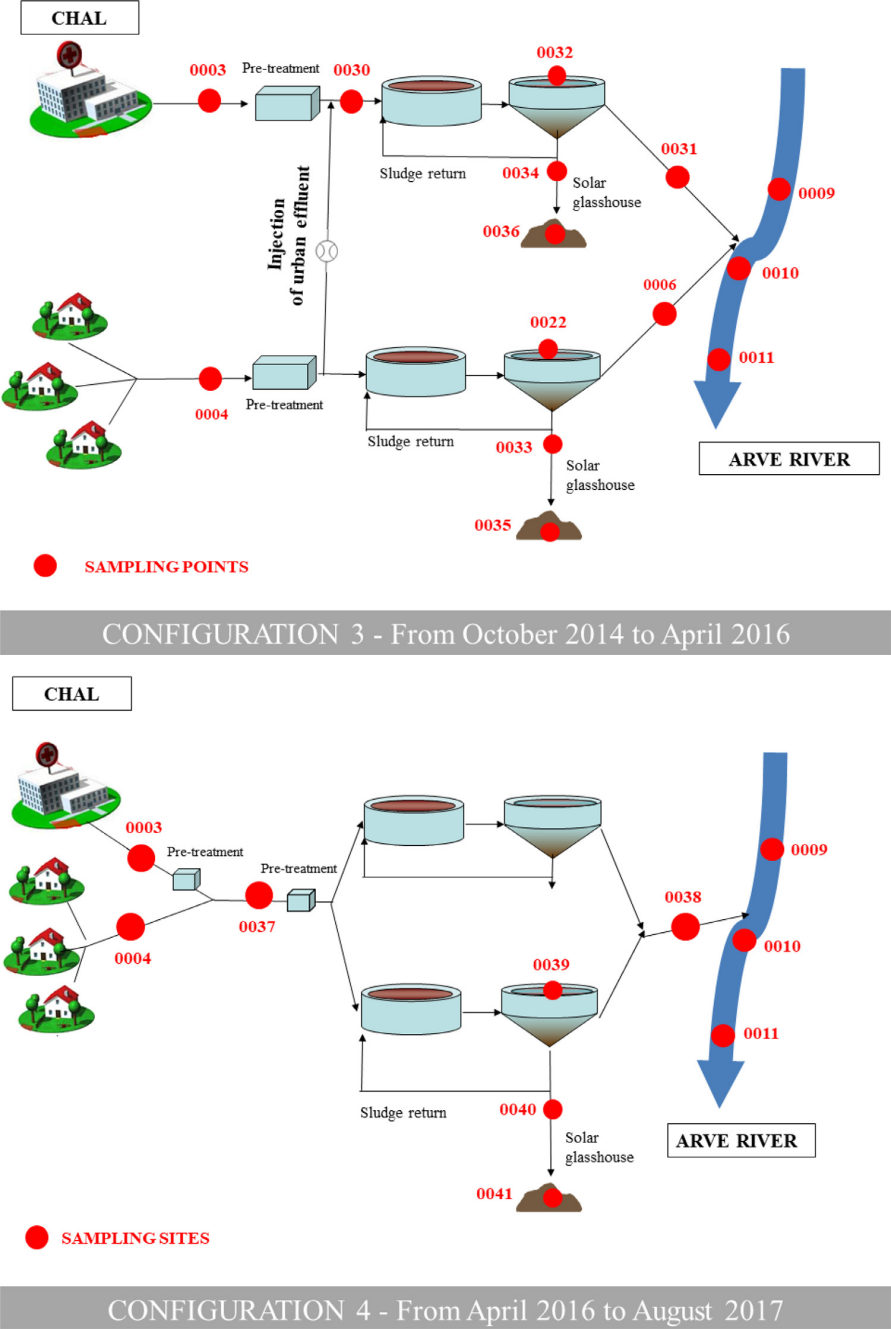
Successive configurations (from top to bottom) of the Bellecombe WWTP from 2011 to 2017 and its sampling points (SPs). See main text for explanations and also Table 1. Figures modified from Chonova *et al*. (2018) and Bergé *et al*. (2018) (references are given in “Related publications”in the “Specifications table”).

The unique configuration of the Bellecombe WWTP with two independent parallel treatment lines provides appropriate conditions for treating and studying HWW and UWW separately. HWW can be treated either mixed with UWW in all three basins, or separately in the dedicated smallest basin (5 400 PE) while UWW is treated in the two other basins. Similarly, the sludge treatment can be carried out separately for the HWW or mixed with the sludge from the UWW (see the successive configurations of the WWTP in Fig. 2).

During the days of experimental campaigns:

• The measured HWW daily volume ranged from 48 to 408 m^3^/d with a mean value of 176 m^3^/d: when treated separately in the 1 280 m^3^ aeration tank, this corresponds on average to a 13.75% hydraulic load and to a 7.3 day residence time.
• The UWW mean daily volume measured during dry and low rain days of experiments ranged from 2 897 to 13 504 m^3^/d, with a mean value of 5 322 m^3^/d corresponding to a 79% hydraulic load and a 1.3 day hydraulic residence time.

For the year 2013, according to internal data provided by the Syndicat des eaux des Rocailles et de Bellecombe (operator of the wastewater treatment plant, partner of SIPIBEL), compared to the dry weather design capacity, the hydraulic load was 81.5% and the pollutant loads were the following ones: 63% for COD, 56% for BOD_5_, 54% for TSS and 51% for TKN.

In addition, supplementary campaigns have also been carried out in sewer systems, to detect and quantify the possible degradation of pharmaceuticals in wastewater during its transfer to the downstream WWTP:

• In the gravity aerobic sewer Choully Galery (UWW), upstream the Geneva WWTP.
• In the anaerobic pressure main from the Pole de Santé hospital (HWW) in Arcachon, France, upstream the Teste-de-Buch WWTP.

### 2.2 Configuration of sampling points

The configuration of sampling points is shown in Fig. 1, Fig. 2 and Table 1.

**Table 1:** GPS coordinates of the SIPIBEL sampling points (SPs).

Sampling sites	ID Sampling Points (SP)	Longitude (decimal format)	Latitude (decimal format)
Bellecombe WWTP	SP_0003 - 0004 - 0005 - 0006 - 0021 - 0022 - 0023 - 0024 – 0025 - 0028 - 0029 - 0030 - 0031 - 0032 - 0033 - 0034 - 0035 - 0036 - 0037 - 0038 - 0039 - 0040 - 0041 - 0042 – 0043 (see Fig. 2 for SPs locations)	6.317500 (SP_0003)	46.13638889 (SP_0003)
Arve upstream	SP_0009	6.318333	46.13694444
Arve downstream 1	SP_0010	6.312222	46.13388889
Arve downstream 2	SP_0011	6.270833	46.15027778
Arve Etrembières	SP_0012	6.231111	46.18305556
Ocybèle WWTP	SP_0007	6.192500	46.17694444
Villette WWTP	SP_0008	6.185278	46.17944444
Arve junction	SP_0013	6.125833	46.20027778
Rhône upstream	SP_0014	6.148056	46.20611111
Rhône Chancy	SP_0015	5.966389	46.14694444
Vessy Station	SP_0016 - 0026 - 0027	6.170833	46.17833333
Veyrier well	SP_0017	6.187778	46.17361111
Carouge well	SP_0018	6.145833	46.18611111
Saconnex well	SP_0019	6.130556	46.16500000
Crache well	SP_0020	6.049167	46.14027778
Choully Galery inlet	SP_0044	6.017817000	46.23295900
Choully Galery outlet	SP_0045	6.040152000	46.21558900
Arcachon hospital outlet	SP_0053	-1.112273000	44.61358600
Arcachon WWTP inlet	SP_0054	-1.129208000	44.60445200

See Fig. 1 map (all SPs except Bellecombe WWTP) and Fig. 2 scheme (Bellecombe WWTP SPs).

The construction of the CHAL hospital (built to replace the former Annemasse hospital located in another catchment) and its separate connection to the Bellecombe WWTP made it possible to sample effluents in different configurations, according to the WWTP evolution and adaptation during the sampling period (Fig. 2). Four configurations of the WWTP have been set during the sampling period:

• Configuration 1 (from 2011 to February 2012, before opening of the CHAL hospital): the Bellecombe WWTP treated only urban wastewater. In addition to samples collected at the WWTP and in the Arve River, wastewater samples were also taken at the former Annemasse hospital (to get prior reference values). This configuration is referred to as the “zero state”.
• Configuration 2 (from February 2012 to September 2014, after opening of the CHAL hospital): the Bellecombe WWTP treated both hospital wastewater (HWW) and urban wastewater (UWW) in two fully separate treatment lines (water and sludge).
• Configuration 3 (from October 2014 to April 2016): a part of the pre-treated UWW was injected into the pre-treated HWW to obtain an “pre-treated mixed effluent”, to be further treated in the HWW treatment line. The average daily volume proportion was approximately 35% HWW/65% UWW.
• Configuration 4 (from April 2016 to August 2017): HWW and UWW were fully mixed at the Bellecombe WWTP inlet and treated in a single treatment line.

### 2.3 Sampling campaigns

Numerous campaigns were carried out at different sites and sampling points indicated in Figs. 1 and 2 (and also in Table 1):

• Urban and hospital wastewater at Bellecombe WWTP:
  ○ 40 campaigns on raw wastewater, treated wastewater, and activated sludge from February 2011 to December 2016 (usually one 24h campaign/month from the opening of the CHAL hospital in February 2012).
  ○ 3 campaigns of seven consecutive days on raw wastewater (June 2013, September 2013 and May 2014).
  ○ 6 hourly step measurement campaigns on raw hospital and/or raw urban wastewater, from 2015 to 2016.
  ○ 20 campaigns on sludges (filter press outlet or solar glasshouse outlet), from April 2015 to August 2017.
• Treated wastewater at Ocybèle WWTP (France) and Villette WWTP (Switzerland): 9 campaigns from 2013 to 2015.
• Arve River and Rhône River: 13 campaigns from 2011 to 2016 (around 3 campaigns/year).
• Vessy groundwater recharge station and Geneva aquifer: 7 campaigns from 2013 to 2015.
• Choully Galery: 5 campaigns on raw wastewater upstream and downstream from 2016 to 2017.
• Arcachon hospital: 1 campaign on raw hospital wastewater upstream and downstream a pressure main (June 2017).

The total number of data (48 439 values) uploaded on Zenodo are detailed in Table 2.

**Table 2:** Number of data.

Samples	Physico-chemical data	Bioassay data	Microbiology data	Hydrobiology data
961	44 455	2 193	1 679	112

### 2.4 Sampling protocol

The aim of the project being to measure trace concentrations of micropollutants, a special attention was devoted to sampling and sample handling processes to ensure the best possible representativeness of every sample analysed in the laboratory. In order to benefit from the experience of previous similar experiments, the SIPIBEL sampling protocol followed the recommendations of the Aquaref Operational Technical Guide [2]:

• 24 h mean samples
  ○ WWTP: volume proportional sampling (150 to 200 elementary samples).
  ○ Surface freshwater: mean sample reconstituted from hourly sub-samples, mixed proportionally to hourly river discharge measurements supplied by EDF utility (electricity provider which regulates the flow in the river) and by Etat de Genève (144 elementary samples: 6/hour).
• Homogenisation and distribution of a sample in flasks: stirring pale, distribution pump, three partial filling of 1/3 of the flasks.
• All equipments in contact with samples are made of glass and Teflon.
• Rinsing of the equipments between every campaign, following a rigorous protocol.
• Blanks to control the reliability of the protocol and validate / correct the measured data.

A log-book was used to report all details and possible incidents during the campaigns and the sampling periods.

### 2.5 Assessment of data quality

Data quality is assessed for each measured value according to the quality of i) the sample, ii) the analysis, and iii) the results of blank samples.

**Fig. 3:**
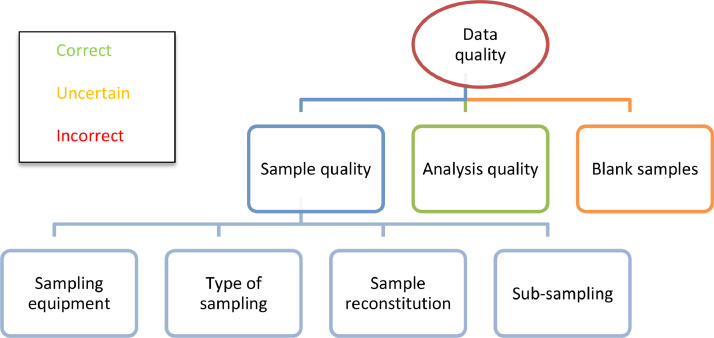
Assessment of data quality.
• Each of the seven above indicators can be declared as “Correct”, “Incorrect” or “Uncertain”. The sample quality is declared:
  ○ Correct, if all seven above indicators are correct.
  ○ Incorrect, if at least one of above indicators is incorrect.
  ○ Uncertain, in any other case.
• Analytical quality is assessed based on the comments given by the laboratories in their analyses reports. For example, the quality is declared “Uncertain” in case of a possible contamination of the sample, a too long period between sampling and analysis (possibly unsatisfactory preservation of the sample before analysis).
• Blank samples make it possible to assess the reliability of the measurement (for example adsorption or desorption phenomena leading to overestimation or underestimation of concentration): the protocol compares the values of blank samples “before” and “after” the sampling and analytical chain, in order to detect and quantify any significant difference beyond measurement uncertainties. Corrections were made if necessary.

• The sample quality is evaluated according to 7 indicators (Fig. 3):
  ○ sampling equipment: conformity (1), rinsing protocol (2).
  ○ type of sampling (3) (discharge proportional, time-proportional, instantaneous grab sample), sampling incidents (4) (non-existing, medium, important).
  ○ reconstitution of mean sample (5).
  ○ sub-sampling: homogenisation (6) and distribution of the sample (7).

Let *x*_1_ and *x*_2_ be the values “before” and “after” the sampling chain, and *u*(*x*_1_) and *u*(*x*_2_) their respective standard uncertainties, as given by the laboratory. It is assumed that the true value of *x_i_* has an approximately 95% probability to be between *x_i_* - 2*u*(*x_i_*) and *x_i_* + 2*u*(*x_i_*) when the *x_i_* values are normally distributed. One calculates the gap *E* between the two values, and its standard uncertainty *u*(*E*):

$$E=\left|x_{1}-x_{2}\right|$$

$$u\left(E\right)=\sqrt{u{\left(x_{1}\right)}^{2}+u{\left(x_{2}\right)}^{2}}$$

One concludes that:

• If *E ≤ 2u*(*E*): the two values are not significantly different and can be considered as equivalent.
• If *E > 2u*(*E*): the two values are significantly different.

Blank checking quality is declared:

• Correct if:
  ○ There is no significant difference between values as explained above.
  ○ The difference is significant but lower than 15% of the measured value of the samples.
• Uncertain if the difference is significant and in the range of 15% to 25% of the measured value of the sample.
• Incorrect if the difference is significant and larger than 25% of the measured value.

Finally, data quality is declared:

• Correct, if sample quality, analysis quality and blank checking quality are all correct.
• Incorrect, if sample quality, analysis quality and/or blank checking quality is/are incorrect.
• Uncertain, in any other case.

During the project, a dedicated database, named DoMinEau and built with Excel files, was established to collect and centralise all data and make them available for all partners of the project. The final values of correct and uncertain data are made publicly available in Zenodo (see Data accessibility in Section 1). Incorrect data have been removed: they correspond to approximately 5% of all data stored in DoMinEau.

## Ethics Statement

The authors declare that the present work did not include experiments on human subjects and/or vertebrates.

## CRediT authorship contribution statement

**Jean-Luc Bertrand-Krajewski:** Data curation, Writing – original draft, Writing – review & editing, Methodology, Funding acquisition. **Rémy Bournique:** Writing – original draft. **Vivien Lecomte:** Project administration, Supervision, Writing – original draft, Methodology, Data curation, Writing – review & editing. **Noémie Pernin:** Writing – review & editing, Data curation. **Laure Wiest:** Writing – original draft, Writing – review & editing, Funding acquisition, Supervision, Formal analysis. **Christine Bazin:** Funding acquisition, Writing – review & editing. **Agnès Bouchez:** Funding acquisition, Writing – review & editing. **Elodie Brelot:** Funding acquisition, Writing – review & editing. **Benoît Cournoyer:** Funding acquisition, Writing – review & editing. **Teofana Chonova:** Funding acquisition, Writing – review & editing. **Christophe Dagot:** Funding acquisition, Writing – review & editing. **Pascal Di Majo:** Funding acquisition, Writing – review & editing. **Adriana Gonzalez-Ospina:** Funding acquisition, Writing – review & editing. **Audrey Klein:** Funding acquisition, Writing – review & editing. **Jérôme Labanowski:** Funding acquisition, Writing – review & editing. **Yves Lévi:** Funding acquisition, Writing – review & editing. **Yves Perrodin:** Funding acquisition, Writing – review & editing. **Sandra Rabello-Vargas:** Funding acquisition, Writing – review & editing. **Liana Reuilly:** Funding acquisition, Writing – review & editing. **Audrey Roch:** Funding acquisition, Writing – review & editing. **Axel Wahl:** Funding acquisition, Writing – review & editing.

## Declaration of Competing Interest

The authors declare that they have no known competing financial interests or personal relationships which have or could be perceived to have influenced the work reported in this article.
